# High Acoustic Impedance and Attenuation Backing for High-Frequency Focused P(VDF-TrFE)-Based Transducers

**DOI:** 10.3390/s23104686

**Published:** 2023-05-12

**Authors:** Sean Toffessi Siewe, Samuel Callé, François Vander Meulen, Damien Valente, Jean-Marc Grégoire, Aline Banquart, Stéphanie Chevalliot, Arnaud Capri, Franck Levassort

**Affiliations:** 1 GREMAN, UMR 7347, University of Tours, CNRS, INSA Centre Val de la Loire, 37200 Tours, France; 2 INSERM Imaging and Brain, UMR 1253, 37000 Tours, France; 3 Carestream Dental France, 77183 Croissy-Beaubourg, France

**Keywords:** transducer, ultrasound, backing, high-frequency, imaging

## Abstract

Backing materials with tailored acoustic properties are beneficial for miniaturized ultrasonic transducer design. Whereas piezoelectric P(VDF-TrFE) films are common elements in high-frequency (>20 MHz) transducer design, their low coupling coefficient limits their sensitivity. Defining a suitable sensitivity–bandwidth trade-off for miniaturized high-frequency applications requires backings with impedances of >25 MRayl and strongly attenuating to account for miniaturized requirements. The motivation of this work is related to several medical applications such as small animal, skin or eye imaging. Simulations showed that increasing the acoustic impedance of the backing from 4.5 to 25 MRayl increases transducer sensitivity by 5 dB but decreases the bandwidth, which nevertheless remains high enough for the targeted applications. In this paper, porous sintered bronze material with spherically shaped grains, size-adapted for 25–30 MHz frequency, was impregnated with tin or epoxy resin to create multiphasic metallic backings. Microstructural characterizations of these new multiphasic composites showed that impregnation was incomplete and that a third air phase was present. The selected composites, *sintered bronze–tin–air* and *sintered bronze–epoxy–air*, at 5–35 MHz characterization, produced attenuation coefficients of 1.2 and >4 dB/mm/MHz and impedances of 32.4 and 26.4 MRayl, respectively. High-impedance composites were adopted as backing (thickness = 2 mm) to fabricate focused single-element P(VDF-TrFE)-based transducers (focal distance = 14 mm). The center frequency was 27 MHz, while the bandwidth at −6 dB was 65% for the *sintered-bronze–tin–air*-based transducer. We evaluated imaging performance using a pulse-echo system on a tungsten wire (diameter = 25 μm) phantom. Images confirmed the viability of integrating these backings in miniaturized transducers for imaging applications.

## 1. Introduction

The backing layer is one of the essential constitutive elements of ultrasonic transducers. It is used as a support for the piezoelectric layer and can be considered as a semi-infinite medium for absorbing echoes from its rear face. This element strongly influences the electro-acoustic response of the transducer and allows for improvements in axial resolution [1], an essential property for imaging. For high-frequency (>20 MHz) imaging applications, the size of the transducer is very often a critical parameter because of the need to operate in restrictive spaces, such as for intravascular ultrasound (IVUS) applications [2] or in a probe head with mechanical scanning (single element transducer) for small animal [3], skin [4] or eye [5] imaging. In such structures, the critical element is usually the backing, the thickness of which in particular can negatively impact its ability to attenuate ultrasonic waves. Several polymers loaded with metallic particles have found use as backing, and their acoustic impedances have been adjusted according to the type of particles and their volume fraction [6,7,8,9,10]. However, these composites often suffer from insufficient acoustic attenuation due to the limited thickness of the backing layer, thus precluding the minimization of this element for the design of high-frequency ultrasonic transducers. Composite mixtures with several phases make it possible to increase acoustic attenuation, such as through the integration of glass bubbles [11]. Regardless, such composite mixing does not correspondingly lead to higher acoustic impedances (typically >10 MRayl). An alternative strategy is to replace the polymer matrix of the composites with materials of higher acoustic impedance. In such cases, ceramics are suitable replacements. The introduction of porosity in the ceramic matrix, such as in yttria-stabilized zirconia (YSZ) or PZT [12], allows the delivery of acoustical impedance higher than 15 MRayl and acoustic attenuation between 0.5 and 4 dB/mm at 2–3 MHz depending on the pore sizes, with reference to the center frequency. The porosity content is usually of the order 25–30%.

Regarding the piezoelectric layer, both the poly(vinylidene fluoride) (PVDF) and poly(vinylidene fluoride-trifluoro ethylene (P(VDF-TrFE)) copolymer have attracted great interest for high-frequency (>20 MHz) applications [13]. These materials are commercially available in thin layers (a few micrometers to tens of micrometers) and can reach resonant frequencies of 20 to 100 MHz. They have low acoustic impedance (close to the propagation medium, i.e., water or biological tissues) while avoiding the use of a matching layer which simplifies the transducer fabrication process. Moreover, their mechanical flexibility allows for simple shaping, such as in device fabricating for direct focusing of a single-element transducer. Previous studies [14,15,16] have specified the design rules for high-frequency transducers integrating these piezoelectric films and demonstrated the influence of several parameters on their performances. They show, in particular, that the acoustic impedance of the backing influences the sensitivity, bandwidth and center frequency of the resultant transducer. To illustrate this influence, we have simulated the electro-acoustic response of a high-frequency single-element transducer in a simple configuration as a function of the acoustic impedance of the backing (hereafter Z_b_). This electro-acoustic response was calculated in water using the KLM model. The KLM model, developed by Krimholtz, Leedom and Mattei [17], is a one-dimensional equivalent electrical circuit based on the same assumption as Mason’s model [18] and allows clear separation of the network of acoustic and electrical ports of the piezoelectric element. Moreover, a matrix formula makes numerical calculation of a multilayer structure integrating a piezoelectric layer easier [19]. Different characteristics of a single-element transducer with one active layer and several passive layers, while taking account of a possible electrical environment, are calculated from different transfer functions, in particular to deduce the electro-acoustic response. The first constitutive element of the transducer is the piezoelectric copolymer P(VDF-TrFE) film, the properties of which appear in Table 1.

These parameters were all deduced from measurements performed on films with a thickness of 18 µm and an active diameter of 6 mm [20]. These geometrical values were retained and employed in the simulation. Ni/Au electrodes on both faces of the piezoelectric film were also simulated (100 nm thick). The electrical environment, in emissions and receptions, is disregarded. The second constitutive element was the backing layer, which we assumed to be a semi-infinite medium capable of avoiding unwanted parasitic echoes coming from the back of the element. Similarly, the glue layer (between the P(VDF-TrFE) film and the backing) was also not considered. These simulations quantify the relative evolution of different transducer parameters as a function of Z_b_—in particular, the sensitivity–bandwidth trade-off of the transducer.

**Figure 1:**
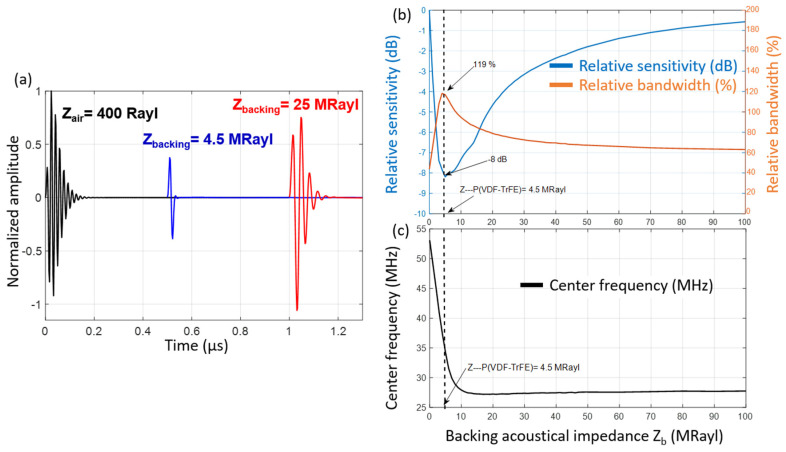
KLM simulation: (**a**) Normalized electro-acoustic responses of the transducer for three Z_b_ values. (**b**) Relative sensitivity in dB (blue line) and relative bandwidth in % (orange line) of the P(VDF-TrFE) (18 µm thick) transducer as a function of the backing acoustic impedance Z_b_ (in MRayl). (**c**) Center frequency (MHz) of the transducer as a function of Z_b_. The reference sensitivity (0 dB) was obtained from the maximum amplitude of the electro-acoustic response in the case of an air backing with Z = 400 Rayl. Other relative sensitivities are derived from ratios of peak-to-peak amplitudes of the calculated electroacoustic responses and peak-to-peak amplitudes of the reference.

Figure 1a shows the normalized electro-acoustic responses of the transducer for three Z_b_ values. The reference sensitivity (0 dB) is obtained from the maximum amplitude (peak-to-peak) of the electro-acoustic response for backing with very low acoustic impedance (Z); in this case, air with Z = 400 Rayl. Relative sensitivities are obtained from ratios of maximum amplitudes of the electro-acoustic responses to the reference. The fractional bandwidth, at −6 dB, is deduced from the transducer frequency response. Figure 1b illustrates the sensitivity–bandwidth trade-off. As one of the two parameters increases, the other decreases as a function of Z_b_. Thus, while an air backing presents the best sensitivity, it simultaneously presents low bandwidth (43.7%). Sensitivity is at its lowest when backing acoustic impedance equals that of P(VDF-TrFE) films (4.5 MRayl) (Figure 1a). Relative sensitivity is 8 dB lower than previous configurations, but relative bandwidth is decidedly higher (119%).

Figure 1c shows the evolution of the transducer center frequency (F_c_) as a function of Z_b_. In the case of an air backing, the center frequency is closer to the anti-resonance frequency of the P(VDF-TrFE) film (Table 1) at 63 MHz, albeit slightly lower with the electrodes, the effect of which is not negligible [14]. For Z_b_ = 4.5 MRayl, the center frequency decreases markedly (F_c_ = 37.4 MHz). A gradual increase of Z_b_ increases sensitivity to reach typically −1 dB at Z_b_ = 80 MRayl. Conversely, bandwidth decreased to 60% and stabilized between 30 MRayl and 100 MRayl. In parallel, the center frequency of the transducer decreases continuously to reach a stable value (F_c_ = 27 MHz, for Z_b_ ≥ 10 MRayl). This decrease in the center frequency stems from the fact that the P(VDF-TrFE) films operate gradually in the λ/2 towards the λ/4 mode. According to the value of the thickness coupling factor of the P(VDF-TrFE) (in our case 27%), which is lower than that of conventional PZT ceramics (typically 57% [21]), sensitivity must be favored and in this case backing with a high acoustic impedance must be retained while maintaining a sufficiently high bandwidth for imaging applications. For example, for Z_b_ = 40 MRayl, the relative sensitivity is −2.3 dB, with a relative bandwidth of 70%, which are sufficient values for our imaging application.

Based on these considerations, metallic substrates are great backing candidates due to their high acoustic impedance values. Moreover, metallic materials exist in several forms (bars, plates, disks) that can act as bottom electrodes for the piezoelectric element. However, metallic substrates are also known for low attenuation [22]. Thus, the fabrication of composite materials for which the matrix and the reinforcements are metals [23,24,25,26] seems well set to offer a suitable backing alternative with increased attenuation while maintaining high acoustic impedance. Moreover, most characterizations of such materials have been conducted at a maximum working frequency of 10 MHz, and specific experimental set-ups and methods for >20 MHz measurements need developing.

The present work describes a new fabrication process and characterization of high-acoustic-impedance and high-attenuation multiphasic metallic backings for high-frequency P(VDF-TrFE)-based transducers. They are designed for medical imaging applications where the sensitivity is of prime importance while keeping minimal bandwidth values. These new backings, with low dimensions, have been integrated into focused single-element transducers, the acoustic and imaging performances of which have, in turn, been characterized.

## 2. Materials and Methods

### 2.1. Specifications

According to simulation results presented in Figure 1, the bandwidth is between 75% and 69.5%, and relative sensitivity varies between −3.7 and −2.3 dB for a backing acoustic impedance of 25 MRayl up to 40 MRayl. These variations are relatively low. The center frequency of the transducer at 27 MHz is also almost stable and retained for our specifications list. Moreover, the range of values of the acoustic impedances represents a good trade-off between stable properties and availability of materials [22]. Because of the flexibility offered by the piezoelectric copolymer P(VDF-TrFE), the backing must also be conformable (cup-shaped) in anticipation of a possible geometric focusing of the resultant transducer. Finally, the backing thickness is fixed at 2 mm to limit the final size of the transducer. This condition imposes a value of the acoustic attenuation coefficient >1 dB/mm/MHz to avoid echoes coming from the reflection of the lower face of the backing.

### 2.2. Fabrication Process of the Multiphasic Backings

Sintered bronze marketed by *AmesPores* [27] served as the base material. These sintered bronze parts can incorporate high-porosity-volume fractions of between 25 and 60%. The manufacturing process of these multiphasic backings uses powder metallurgy technology. This technology consists of compacting spherical metal powders (bronze in our case) in molds or tooling with the negative shape of the required part and then sintering the resulting preform. Such preforms can be created by gravity filling, uniaxial compaction, isostatic compaction or extrusion, depending on the material to form, the porosity level required and the geometrical shape of the component. Sintering involves heating the preform to a temperature below the melting point of the base metal (for bronze, this is between 700 °C and 1300 °C) in a controlled atmosphere for a prescribed time. The heat causes the grains to weld together to form a cohesion of the part [27]. The product output of this process is a structurally functional part with a controlled level of microporosity.

The interest of this technology for our study is, on the one hand, the choice of the porosity content and, on the other hand, the size of the metallic powder, which—in agreement with the center frequency of our study—is of the order 25–30 MHz. Moreover, the acoustic attenuation coefficient of this material allows for optimization, provided the ultrasound wavelength lies in the same range as the microstructural dimensional characteristics either in the grain size or the pore size. Therefore, we used sintered bronze disk shapes with an average grain size of 100 μm, an initial dimension of 8 mm for the external diameter and a thickness of 10 mm. The porosity rate is 35%, with an average pore size of 53 µm and a maximum pore size of 139 µm. The grain and pore sizes of these materials are consistent with the value of the wavelengths used (144–173 µm) according to the longitudinal velocity in the multiphasic backing.

The first attempts to directly bond piezoelectric films of P(VDF-TrFE) on these sintered bronze cylinders (on a flat surface) with an adhesive epoxy resin layer were not satisfactory due to inadequate contact with the pore at the surface of the material. Similarly, conventional cup-shaping using a metallic ball pressed into the upper face for transducer focusing led to an uncontrolled deformation of the material (bulging of the part). In the following sections, these sintered bronze cylinders are named *sintered bronze–air* (to account for the porosity of this material). To overcome these bonding and shaping difficulties, impregnation with two different materials—tin and epoxy resin—was implemented. The two impregnation processes used differed in specific ways. 

Tin is chosen for the first impregnation process because it is a ductile metal with a low melting temperature of 232 °C. This low melting temperature makes it possible to impregnate metals with high melting temperatures, such as bronze (890 °C). During the tinning process, molten tin penetration is achieved at temperatures around 400 °C using a soldering iron. The method involves the introduction of the sintered bronze disc into a tinning flux (LOCTITE HYDX-20 Liquid Flux [28]), followed by degassing the whole assembly for 5 min. Fluxing facilitates the deposition and adhesion of the tin. The tin is lead-free solder (Sn 96% Ag 4% SAC305) with a melting temperature of 232 °C. With the tip of the soldering iron positioned on the surface of the sintered bronze cylinder, the temperature rise of the *sintered bronze–air* cylinder allows the solder (tin) to penetrate inside the cylinder by thermal conduction. The molten tin fills the cylinder by capillary action through the pores, although we could not entirely discount the existence of empty pores. The removal of flux residues involves using an ethanol solution.

In the second process, filling the *sintered bronze–air* with the epoxy resin takes place, with a vacuum degassing of the assembly for 10 min. During this process, the air inside the porous cylinder escapes, making room for the epoxy resin. This assembly is then heated for 1 h at 65 °C in an oven to induce polymerization of the epoxy resin. In the rest of this article, the *sintered bronze–air* cylinders impregnated with epoxy resin are termed *sintered bronze–epoxy–air* and *sintered bronze–tin–air* for those with tin (to account for the residual porosity in both cases).

**Figure 2:**
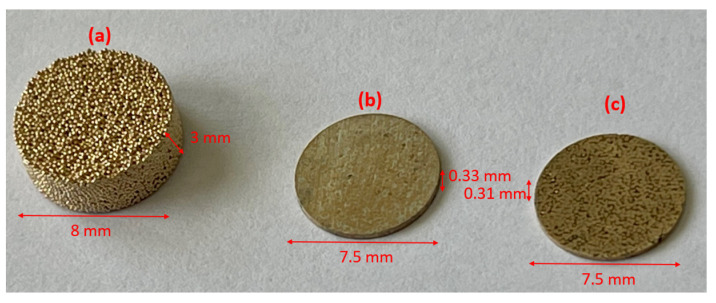
Photographic image of the three materials, (**a**) sintered bronze–air, (**b**) *sintered bronze–tin–air* and (**c**) *sintered bronze–epoxy–air*, with indicative dimensions (diameters and thicknesses).

Figure 2 shows a photograph of the three obtained multiphasic materials (disk shape). Samples were machined to reduce their dimensions, especially the thickness, allowing us to correctly measure their acoustic properties (described in Section 3.2). For the corresponding experimental set-up, the diameter needs to be slightly higher than the active diameter of the transducer (5 mm) used for the acoustic characterization. A diameter of 7.5 mm was retained. It is important to note that the thickness of the *sintered bronze–air* samples was not easy to reduce because this material is brittle and difficult to machine, owing to the porosity content and frequent breakage. Five *sintered bronze–tin–air* and two *sintered bronze–epoxy–air* samples were obtained after the machining process. All sample thicknesses are specified (Table 2) in the following section. The *sintered bronze–epoxy–air* material is similarly difficult to machine, which explains the lower number of available samples compared to the *sintered bronze–tin–air* sample.

### 2.3. Microstructural Observations of the Multiphasic Backings

**Figure 3:**
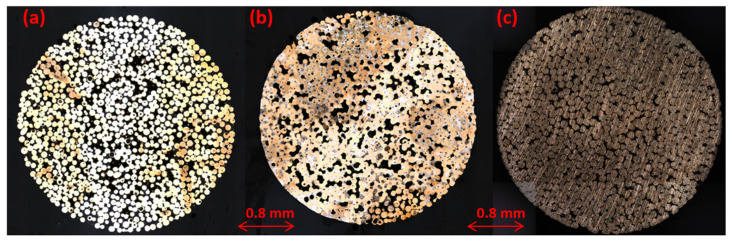
Optical microscopic surface images of (**a**) *sintered bronze–air*, (**b**) *sintered bronze–tin–air* and (**c**) *sintered bronze–epoxy–air* disks. Air and epoxy resin are fully inter-mixed for the *sintered bronze–epoxy–air* sample due to sample coating preparation. Yellow color: bronze, gray color: tin, black color: air, and intermixed air and resin for (**c**). The difference in the color of the bronze between (**a**–**c**) is related to aberrations during image acquisition.

The different phases in the samples were visually analyzed (observed) using an optical microscope (OLS4000-Olympus). Samples were encapsulated in epoxy resin and polished prior. Figure 3 shows microscopic images of the surface of the three representative samples. The two phases of the *sintered bronze–air*, and the three phases of the *sintered bronze–tin–air*, are identifiable. For *sintered bronze–epoxy–air*, the epoxy resin and air phases are mixed up, in part due to the preparation of the coatings. This preparation leads to a migration of the resin in the cavities (which are initially empty).

**Figure 4:**
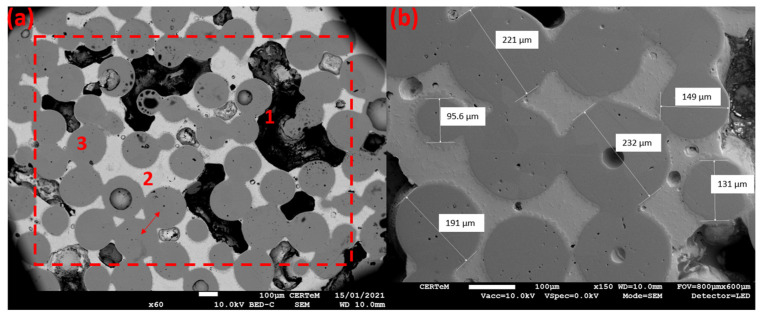
SEM image of the *sintered bronze–tin–air* sample. Three phases are identifiable in (**a**); 1: epoxy resin (black/carbon), 2: tin (light grey), 3: bronze (dark gray). The red arrow illustrates the interpenetration between bronze grains. The dashed red lines delimit the area used for the numerical modeling (wave propagation, Figure 5). (**b**) Selected dimensional measurements of several bronze spheres in the *sintered bronze–tin–air* sample at ×150 magnification.

Scanning electron microscope (SEM –JEOL JSM-7900F) observations confirmed the presence of three phases in the *sintered bronze–tin–air* material (Figure 4a). The sample preparation induces the resin (black coloring in Figure 4a) to migrate into the pores, which would otherwise be empty. The images also show interpenetration between the bronze grains (illustrated by a red arrow in Figure 4a). This phenomenon is called “neck formation” and is well-documented in the literature [29] and caused by high temperatures during the sintering process of the material. According to Figure 4a, the surface fractions of sintered bronze, tin and air are 61%, 18% and 21%, respectively.

The bronze grain sizes (between 100 and 250 µm) could also be measured, as shown in Figure 4b. These values all fall in the range required for our study.

## 3. Results and Discussion

This section involves three parts. The first is concerned with modeling ultrasonic wave propagation through our multiphase materials to quantify the phenomenon of acoustic attenuation. The second is concerned with the experimental measurements of these attenuations. The third part concerns itself with the integration of these backings in ultrasonic transducers, which are then characterized and used for the realization of wire phantom images to confirm the goodness of properties of the new materials used for backing composites.

### 3.1. Theoretical Acoustic Attenuation 

#### 3.1.1. Pseudo-Spectral Time-Domain (PSTD) Method Propagation Model

A pseudo-spectral time-domain (PSTD) numerical model adapted to our specific configurations with biphasic or triphasic media was employed. The pseudo-spectral method, introduced by Kreiss and Oliger in 1972 [30], is an alternative to the finite element and finite difference methods. It involves calculating spatial derivative functions defined on a uniform Cartesian spatial grid in the frequency domain. The propagation wave equation is in this study considered as a bi-dimensional heterogeneous fluid, and shear waves are deliberately neglected [31]. A staggered spatial grid reduced numerical artifacts. Temporal implementation relied on the staggering fourth-order Adams–Bashforth method [32]. In the case of acoustic wave propagation over distances of several wavelengths, the PSTD method requires a few nodes per wavelength to exhibit high numerical stability when compared to finite difference or finite element methods. In the case of wave propagation in media with low acoustic velocity and density contrasts, the Fourier pseudo-spectral method requires only two nodes per minimum wavelength to provide exact spatial derivatives [33]. To minimize reflections on the numerical grid boundaries and counter the wrap-around effect from the Fast Fourier Transform (FFT), PSTD methods classically used matched absorbing boundary conditions called Perfectly Matched Layers (PML) [34].

**Figure 5:**
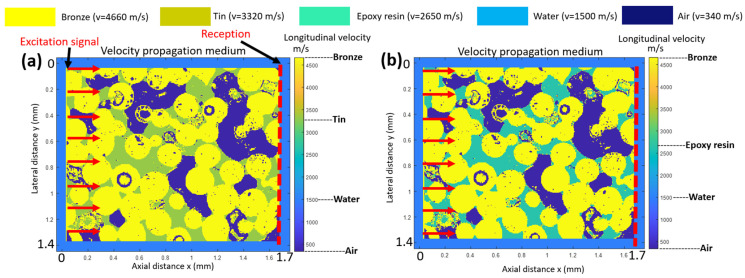
Maps of the spatial distribution of the longitudinal wave velocities used for the PSTD simulations: (**a**) *sintered bronze–epoxy–air* and (**b**) *sintered bronze–resin–air* backings.

Using the image obtained with the SEM (Figure 4), the starting point was to perform a conversion of the image obtained (jpeg format) into a matrix of points for the MATLAB software [35]. Only three amplitudes, or gray levels, are retained for the conversion. These three amplitudes correspond to the three phases of the material: bronze, tin and epoxy resin or air. Figure 5a,b present a colormap of the longitudinal wave velocities of the constitutive materials for the *sintered bronze–tin–air* and *sintered bronze–epoxy–air* samples, respectively. In Figure 5b, the spatial arrangement is the same as that in Figure 5a, with the only change being the replacement of the tin with the epoxy resin phase. Water surrounds the backing. In this 2D model, the grid size is 298 points on the lateral dimension and 360 points on the axial dimension. An eight-node PML is employed. The spatial increments used are ∆x = ∆y = 5 µm, which are inferior to the wavelength of the excitation signal (133 µm). At ∆t = 0.1 ns, the temporal increment was in agreement with the stability condition of Courant [36], thus ensuring that the numerical model produces a coherent solution when solving the system of partial differential equations. The attenuation from each phase of the multiphasic material is not individually taken into account to focus only on the scattering effects. The excitation signal is a sinusoidal signal with two periods and a frequency of 30 MHz. This frequency agreed with the specifications defined for the center frequency of the transducer. The excitation condition is applied over the entire height (1.3 mm, red arrows) of the backing, and the waves propagate across the length (1.7 mm, red dashed line) of the calculation grid. 

#### 3.1.2. Theoretical Acoustic Properties in the Developed Multiphase Backing Materials

**Figure 6:**
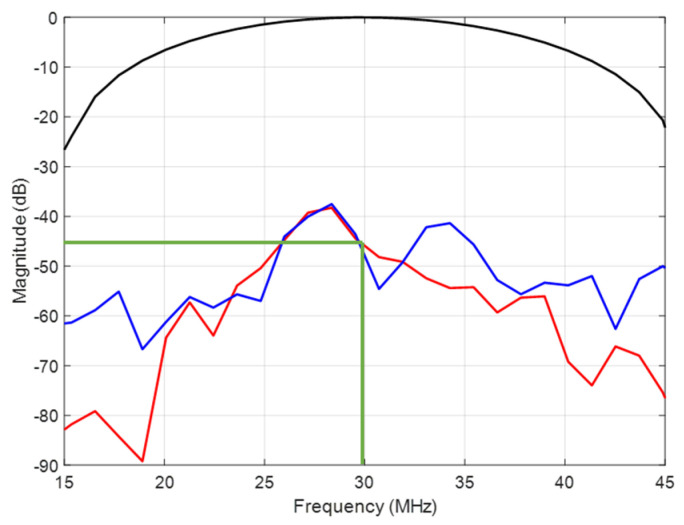
Simulated normalized spectra of the excitation signal (black line) and the relative received signals for *sintered bronze–epoxy–air* (red line) and *sintered bronze–tin–air* (blue line) backings at 1.7 mm length.

Attenuation due to scattering in the backing was deduced from the spectral ratio of the excitation signal to the same signal after propagation over the backing length (1.7 mm, vertical red dashed line). Figure 6 compares the spectra of the excitation signal and the same signals after acoustic propagation (1.7 mm) for the two backing configurations. Based on the same excitation signal, similar attenuations of about 45 dB at 30 MHz are obtained (green line, Figure 6) for the *sintered bronze–epoxy–air* (red line) and *sintered bronze–tin–air* (blue line) backing materials. The corresponding acoustic attenuation coefficient is 0.88 dB/mm/MHz if a linear behavior as a function of frequency is assumed. The longitudinal wave velocity deduced from these simulations was 4000 m/s for both backings (the ratio of the backing length to the time of flight).

These attenuation values are satisfactory and concordant with the specifications defined above. The value of the acoustic attenuation coefficient was slightly lower than our objective target of 1 dB/mm/MHz, but our simulations did not take due account of the intrinsic attenuation of the different phases (bronze, tin, epoxy resin). Nonetheless, this value is comparable to attenuation values found in the literature for a multiphasic metallic medium [23,24,25,37].

The following part is devoted to experimental acoustic properties measurements, allowing us to compare them with those obtained theoretically.

### 3.2. Experimental Acoustic Properties of Multiphasic Materials

**Figure 7:**
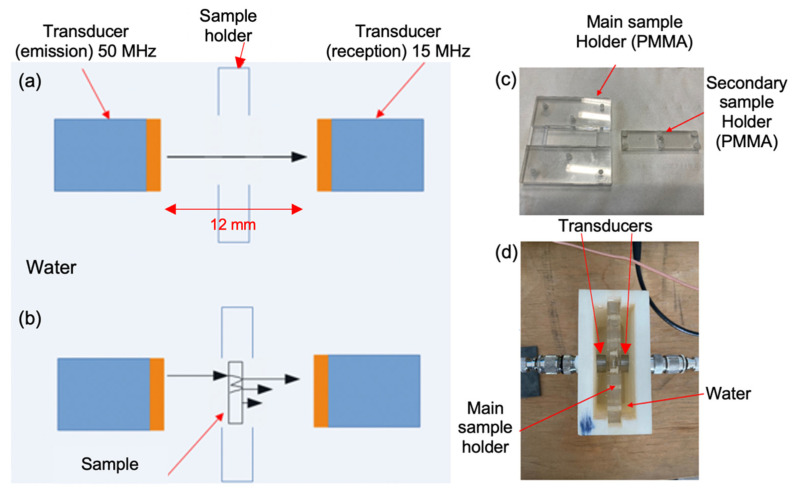
(**a**) Scheme of the experimental set-up in water without the sample. (**b**) Scheme of the experimental set-up with the sample. (**c**) Photograph of the sample holder (without sample). (**d**) Top view of the experimental set-up with a sample inside.

Most acoustic characterizations of multiphasic materials are performed at a maximum frequency of 10 MHz [23,25,37]. Only a few experimental set-ups have involved high-frequency characterizations [38]. Here, a set-up based on the insertion/substitution method was developed [39,40] to measure the acoustic properties (velocity and attenuation). Using two transducers (transmitter/receiver), this transmission method consists of making an initial reference measurement through a known water thickness (12 mm, Figure 7a). Test measurements are performed after replacing the reference volume with a similarly sized test sample placed between the two transducers under a water medium (Figure 7b). Special attention is paid to the perpendicular alignment of the test samples against the acoustic beam [41], thus guaranteeing the repeatability of the measurements. The fabricated small tank contains a sample holder which consists of two parts (Figure 7c). The second is removable and allows the sample to be characterized to be inserted into the first main part. Then, the assembly is placed in the tank perpendicular to the acoustic beam (Figure 7d). Here, an AVG-3-C pulse generator (AVTECH, Taipei, Taiwan) drove the plane transducer with a center frequency of 50 MHz in a transmit mode. In reception, the plane transducer used had a center frequency of 15 MHz. The lower frequency utilized here was chosen because of the high acoustic attenuation of these samples in water, which adequately modifies the spectral components of the transmitted signal. This way, sufficient sensitivity is preserved to extract representative signals. The two transducers had an active diameter of 5 mm. The received signals were visualized and recorded on an RTM3004 5GSa/s digital oscilloscope (Rodhe & Schwarz GmbH, Munich, Germany). The times of flight for the reference and transmitted signal are measured by thresholding. Differences in times of flight for the reference and test measurements allow for the deduction of longitudinal wave velocity (using the thicknesses of the samples). We did not use an inter-correlation method to determine the longitudinal wave velocity because the characteristics of the two signals (i.e., the reference and the transmit signal through the sample) were different. From the longitudinal wave velocities, the acoustic impedance of the sample is obtained. This enables the determination of the transmission coefficients from water to sample and from the sample to water. Then, the ratio of the modulus spectra over the signal transmitted through the test sample to the modulus spectra of the reference signal was used to deduce attenuation, taking the transmission coefficients into account [39,40].

The measured sample thicknesses were adjusted to ensure that the transmit signals employed were of sufficient amplitude. Accordingly, the working thicknesses of the samples at all compositions were between 0.3 and 1 mm. Table 2 provides the geometric characteristics and the number of replicate samples used for each material composition. All backing samples had a diameter of 7.5 mm. Sintered bronze–air samples were excluded entirely from this characterization because reaching thicknesses of <1 mm by machining without unduly degrading these samples proved unfeasible. Dense bronze and brass samples were similarly characterized using the same procedure to better compare the deduced properties against the literature values on similar materials.

**Figure 8:**
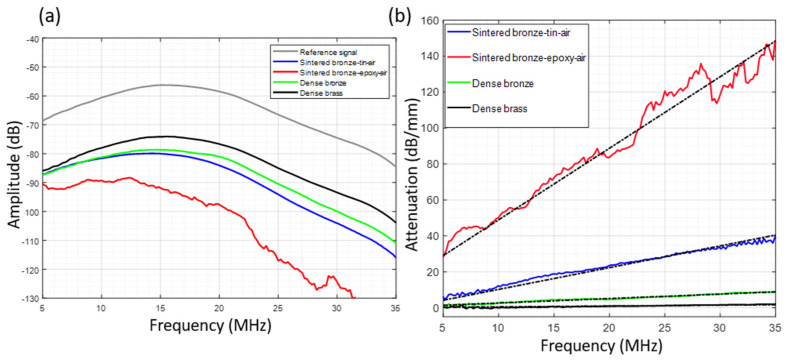
(**a**) Frequency response of the reference signal (gray), *sintered bronze–tin–air* (blue), *sintered bronze–epoxy–air* (red), dense bronze (green) and dense brass (black) samples. (**b**) Acoustic attenuation as a function of frequency for *sintered bronze–tin–air* (blue), *sintered bronze–epoxy–air* (red), dense bronze (green) and dense brass (black) samples. A linear regression (black dotted lines) is performed on the data to extract attenuation coefficients in dB/mm/MHz.

Figure 8a shows the frequency responses from the reference signal and the characterized materials. We observe that the maximum amplitudes are around 15 MHz and are consistent with the transducer bandwidth in reception (the minimum frequency and the maximum frequency at −6 dB are 9 MHz and 22.6 MHz, respectively). Figure 8b shows the attenuation (in dB/mm) as a function of frequency (between 5 and 35 MHz) for the four characterized materials. This figure presents the most attenuating samples of each material. We observe that the *sintered bronze–epoxy–air* sample is the most attenuating material among the samples studied. For this material, variations of the acoustic attenuation around the mean linear behavior (dashed black line, Figure 8b) are more important than for the other materials. This is mainly due to the low signal-to-noise ratio of the transmitted signal. 

Table 2 summarizes all the averaged values obtained for the number of samples machined for each composition. The density of each studied sample was measured using Archimedes’ method. The longitudinal wave velocity measured for dense brass agreed with the literature [22] and thus validated the measurement set-up. The acoustic attenuation coefficient of the *sintered bronze–tin–air* backing materials presented was the mean of five samples (1.2 dB/mm/MHz) with a standard deviation of 0.036 dB/mm/MHz. These values show a variation of the attenuation due to the manufacturing process of this material. The average value of the acoustic impedance was 32 MRayl, with a standard deviation of 2.27 MRayl. For the *sintered bronze–epoxy–air* material, the mean value of attenuation was obtained on two samples whose attenuation was ≥100 dB/mm at 25 MHz, corresponding to an acoustic attenuation coefficient of >4 dB/mm/MHz. The average acoustic impedance was 25.5 ± 3 MRayl. Compared to the theoretical values obtained previously (0.8 dB/mm/MHz for both backings), the measured attenuation values were higher. At 1.2 and >4 dB/mm/MHz for *sintered bronze–tin–air* and *sintered bronze–epoxy–air*, respectively, this difference can be explained by the fact that, in the simulations with the PSTD model, intrinsic attenuation from these materials was overlooked. Regarding the longitudinal wave velocity, the measured value for *sintered bronze–tin–air* samples was 4320 m/s and around 4600 m/s for *sintered bronze–epoxy–air* samples. These velocities are slightly higher than the theoretical values (around 4000 m/s) resulting from the previous simulations. Contrary to the simulations, during velocity measurement, the samples are immersed and have a part with open porosity in which water can penetrate, which invariably modifies the velocity values. Moreover, the measured velocities of the two backings are very close to that of the dense bronze. This closeness is consistent with the fact that the bronze spheres of the materials studied are consolidated in three dimensions. Thus, the velocity values of the two backings are highly dependent on the velocity of dense bronze, regardless of the impregnation (tin or epoxy resin) in the backing material. However, the acoustic attenuation values in these impregnation materials strongly influence that of the final material. Indeed, the attenuation of epoxy resin is much greater than that of tin at high frequency and contributes significantly to attenuation increases of the *sintered bronze–epoxy–air* backing.

The two materials offer high attenuations and slightly different acoustic impedances due to their different densities. These differences present differing bandwidth–sensitivity trade-offs for integration into high-frequency transducers. With the acoustic impedances of the two materials (Table 2), while using similar characteristics of the single-element transducers simulated in the introduction of this article, the two corresponding transducers recorded similar matching theoretical center frequencies at 27.3 MHz. With the *sintered bronze–tin–air* and *sintered bronze–epoxy–air* backings, a relative bandwidth at −6 dB of 72% and 75% and a relative sensitivity (compared to an air backing) of −3 dB and −3.6 dB were calculated from KLM simulations, respectively. The following section presents the experimental evaluation of the performances of these two multiphase materials used as backings for a high-frequency P(VDF-TrFE)-based transducer.

### 3.3. Transducer Integration and Imaging

#### 3.3.1. Transducer Fabrication

**Figure 9:**
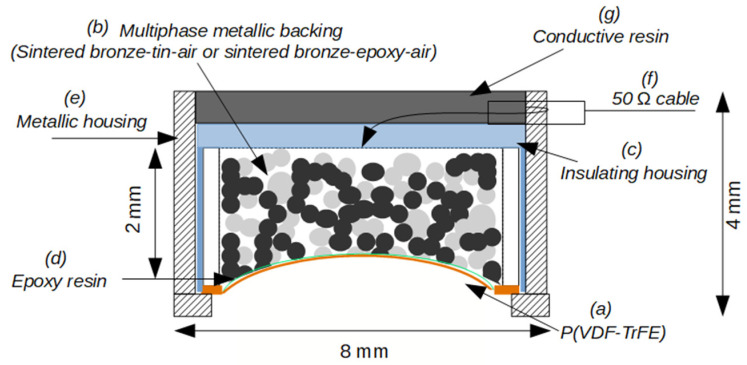
Scheme (cross-section) of the high-frequency focused P(VDF-TrFE)-based transducer.

Figure 9 illustrates the cross-section of the fabricated transducer. P(VDF-TrFE) films (18 µm thick) from PolyK Technologies, Pennsylvania, USA [42] were used in the fabrication of two transducers. The electromechanical properties and geometrical characteristics of this piezoelectric film [17,20] appear in Table 1. The fabrication process starts with a cylinder of 2 mm thick *sintered bronze–tin–air* (or *sintered bronze–epoxy–air*). This cylinder is curved on its top face using a steel ball with applied pressure to achieve the focusing aperture. The steel ball radius defines the focal distance of the transducer (14 mm). After curving, the backing layer (b) is incorporated in an insulation housing (c), and a piezoelectric copolymer (a) is bonded onto the front of (b) by using EPOTEK 301 epoxy resin (d) from Epoxy Technology, Billerica, USA. Epoxy bonding thickness <3 µm was sufficient to bond (a) and (b). Into a metallic housing (e) was inserted the (a-b-c-d) multilayer, which also served to establish electrical contact with the top electrode of the P(VDF-TrFE) film. To the rear face of the conductive backing is connected a 50 Ω coaxial cable [43] (f). Finally, a conductive resin (g) was applied to improve the transducer shielding and sealing. The external final dimensions of the transducer after assembly were 8 mm diameter and a thickness of 4 mm.

#### 3.3.2. Experimental Characterization

**Figure 10:**
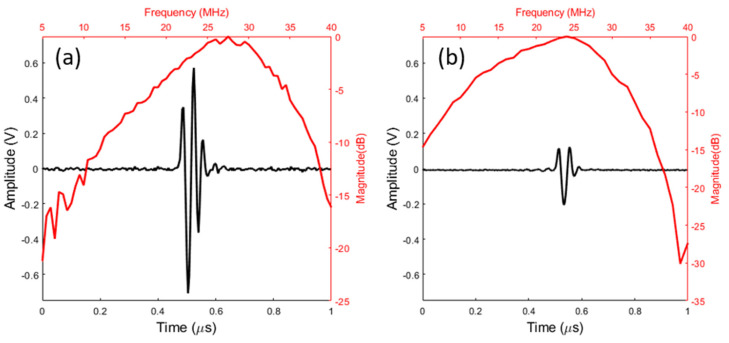
Electro-acoustic responses of the (**a**) *sintered-bronze–tin–air-* and (**b**) *sintered-bronze–epoxy–air*-based transducers (black: temporal response; red: frequency response).

We measured the electro-acoustic response of the two transducers at their focal distance using a steel plane target placed in water. We used a homemade pulse receiver [44] for the characterizations. The experimental conditions for comparing the electro-acoustic performances of the two transducers were identical. Figure 10 presents the electro-acoustic responses for the *sintered-bronze–tin–air*-based transducer (Figure 10a) and the *sintered-bronze–epoxy–air*-based transducer (Figure 10b). The two transducers have the same focal distance (14 mm) and the same f-number (2). This f-number (ratio of the focal length to the active diameter of the piezoelectric film) was chosen for imaging application purposes, for which typical values are between 2 and 3. The center frequency of the *sintered-bronze–tin–air*-based transducer was 27 MHz, and the relative bandwidth at −6 dB was 65%. The center frequency was deduced from the mean value of the bandwidth at -6dB. These results are close to those obtained theoretically. For the *sintered-bronze–epoxy–air*-based transducer, the center frequency was lower at 21.5 MHz, while the relative bandwidth at −6 dB was higher (97%). The latter’s sensitivity was significantly lower (12 dB lower) than that of the earlier (*sintered-bronze–tin–air*-based) transducer. The thickness of the glue layer (epoxy resin) was measured from a metallographic analysis (cross-section) of a transducer manufactured using the same process with a sintered-bronze–tin–air backing. The layer has a thickness of 1 µm with good uniformity. This low value has no influence on the behavior of the transducer (electro-acoustic response), as was previously verified using a dense and flat brass backing on which the same P(VDF-TrFE) film was glued with the same pressure. For this configuration, the modeling with the KLM scheme was performed and the glue layer has no influence up to a thickness of about 3 μm. For the second transducer with *sintered bronze–epoxy–air* backing, the porosity of the sintered bronze has previously been partially filled with epoxy resin, and locally—with the addition of the glue layer—the total thickness is higher than 3 μm and can modify the values of sensitivity (which decreases) and bandwidth (which increases). This is what is measured globally on the electro-acoustic response. We deduced axial resolutions from the electro-acoustic responses. The *sintered-bronze–epoxy–air*-based transducer had a better axial resolution of 30 µm, compared to 50 µm for the *sintered bronze–tin–air* at higher bandwidth. These properties appear in Table 3. As specified in the introduction, the sensitivity–bandwidth trade-off must favor sensitivity while not reducing the bandwidth inordinately; the *sintered bronze–tin–air* backing is thus retained for transducer fabrication.

#### 3.3.3. Phantom Imaging

**Figure 11:**
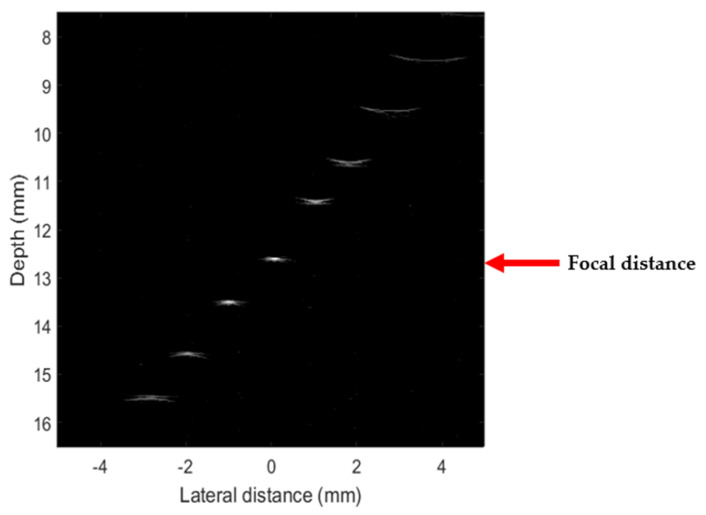
B-mode images of the tungsten wire phantom obtained with the *sintered-bronze–tin–air*-based transducer. The diameter of the wires is 25 µm.

A *sintered bronze–tin–air* backing was used to fabricate another transducer with a 12.7 mm focal distance (corresponding to a f-number of 2.1). The imaging performance of this transducer was evaluated using a wire phantom. The phantom comprised eight tungsten wires with a diameter of 25 µm. This diameter was inferior to the imaging wavelength in water (55 µm), which allowed us to deduce the lateral resolution [45]. The wires were axially positioned and linearly spaced with a vertical and horizontal spacing of 1 mm. We connected the test transducer to a lab-made transceiver module. The pulse excitations had an amplitude of −179 V (U_e_), with a width at half-height of 7 ns. We passed the signals received by the transducer through a receiving circuit whose amplification was set at 26 dB and digitized at a sampling frequency of 200 MHz by a 12-bit Acqiris DP310 data acquisition card (Agilent Technologies, Santa Clara, CA, USA). We deduced insertion losses (𝐼𝐿) for the transducer from the relation:

IL = 20 × log_10_(U_e_/U_r_),

where U_e_ and U_r_ are the excitation and reception peak voltages evaluated from the received signal in the pulse-echo mode. An IL value of 46 dB for a *sintered-bronze–tin–air*-based transducer was deduced. During imaging, the *sintered-bronze–tin–air*-based transducer was linearly translated in a lateral direction to obtain a B-scan image by using a motorized stage. Figure 11 shows the acquired B-mode images of the tungsten wire phantom with 40 dB of dynamic range. We observed low-level sidelobes of the wire outside the focal zone. The full width at half-maximum (FWHM) for the wire placed at the focal distance was equal to 125 µm and corresponded to the lateral resolution. This value is in good agreement with the theoretical value (R*_lateral_* = 119 µm), calculated using the following Equation [45]:

R_𝑙𝑎𝑡𝑒𝑟𝑎𝑙_ = 1.028 × λ × f-number.

Round-trip echoes were not identified during electro-acoustic response measurements in Figure 10a and confirmed by B-mode image (Figure 11).

## 4. Conclusions

This paper described a fabrication process and corresponding acoustical properties of high-attenuation and high-acoustic-impedance multiphasic backings based on sintered bronze for high-frequency P(VDF-TrFE)-based transducers. A feature of the fabrication process was the impregnation of a porous metallic matrix, such as that of sintered bronze, with another metal (tin). Due to incomplete impregnation, the unfilled air cavities within the matrix lead to a triphasic *sintered bronze–tin–air* composite. A similar composite, *sintered bronze–epoxy–air*, was similarly fabricated for comparison by replacing tin with epoxy resin. The measured acoustic attenuation coefficient for the *sintered bronze–tin–air* backing was 1.2 dB/mm/MHz with acoustic impedance of 32.4 MRayl, and was superior to the 4 dB/mm/MHz for the *sintered bronze–epoxy–air* backing (25.5 MRayl). A dedicated 2D numerical study (PSTD) quantified acoustic attenuation induced by scattering through the two composites. Our results confirmed that the presence of unfilled air cavities was significantly crucial to the increase in attenuation. Moreover, the mean grain sizes of the sintered bronze spheres employed must be judiciously chosen to ensure the center frequency of the transducer integrating these composites as backing duly maximizes attenuation. 

A preliminary study with the KLM scheme showed that it was feasible to use backings with high acoustic impedance (typically beyond 25 MRayl) to favor the sensitivity of the P(VDF-TrFE)-based transducers while keeping a sufficient bandwidth value for imaging applications. High-frequency P(VDF-TrFE)-based transducers have been fabricated with the two backings (2 mm thick). We have characterized these two focused single-element transducers. Echoes from the rear of the backings were not observed on the pulse-echo measurements, which confirmed that these materials were useable at a reduced size. The center frequency and −6 dB fractional bandwidth for the *sintered-bronze–tin–air*-based transducer were 27 MHz and 65%, respectively. Although the *sintered bronze–epoxy–air* backing exhibited higher attenuation performance, the *sintered-bronze–tin–air*-based transducer was retained due to its higher sensitivity (+12 dB). Finally, B-mode imaging of a phantom comprising 25 µm-diameter tungsten wires, performed using a *sintered-bronze–tin–air*-based transducer, showed consistent performances in terms of dynamic range (40 dB), axial resolution (50 µm) and lateral resolution (125 µm). 

These results support our translational objective of integrating these new metallic composites with high acoustic attenuations and impedances into miniaturized high-frequency P(VDF-TrFE)-based transducers.

## Figures and Tables

**Table 1:** Electromechanical properties of P(VDF-TrFE), used to calculate electro-acoustic responses of a single-element transducer with the KLM scheme.

Properties	Value
Active diameter	6 mm
Thickness	18 µm
Density(ρ)	1890 kg/m^3^
Longitudinal wave velocity (v_l_)	2370 m/s
Anti-resonance frequency in the air (f_a_)	63 MHz
Electromechanical coupling coefficient (k_t_)	27%
Relative dielectric permittivity (ε_33_^s^/ε_0_)	4.8
Mechanical loss tangent (tan δ_m_)	4%
Electrical loss tangent (tan δ_e_)	12%

**Table 2:** Measured acoustic properties of the samples (SB: sintered bronze). All values are mean (averaged over the total number of the samples tested). Nt: Number of measured samples, Tb: sample thickness, ρ: density, v: longitudinal wave velocity, Z: acoustic impedance, α: acoustic attenuation coefficient.

	SB Tin-Air	SB Epoxy-Air	Dense Bronze	Dense Brass
Nt	5	2	1	1
Tb (mm)	0.53–1–0.33–0.4–0.58	0.31–0.42	0.96	0.97
ρ (kg/m^3^)	7509 ± 526	5745 ± 747	9478	8576
v (m/s)	4320 ± 173	4596 ± 276	4722	4755
Z (MRayl)	32 ± 4	25.5 ± 3	44.8	40.8
α (dB/mm/MHz)	1.2 ± 0.036	>4	0.24	0.04

**Table 3:** Experimental electro-acoustic performances of the *sintered-bronze–tin–air*-based and *sintered-bronze–epoxy–air*-based transducers. Fc: center frequency; BW_−6 dB_: fractional bandwidth at −6 dB; R_ax_: axial resolution (at −6 dB); F_d_; focal distance; F-number: active diameter/focal distance; A_pp_: normalized amplitude to the peak-to-peak amplitude of the *sintered-bronze–tin–air*-based transducer.

	*Sintered Bronze–Tin–Air*	*Sintered Bronze–Epoxy–Air*
Fc (MHz)	27	21.5
B_−6dB_ (%)	65	97
R_ax−6dB_ (µm)	50	30
F_d_ (mm)	14	14
F-number	2	2
A_pp_ (dB)	0	−12

## Data Availability

Not applicable.
